# Iron Deposition in Brain: Does Aging Matter?

**DOI:** 10.3390/ijms231710018

**Published:** 2022-09-02

**Authors:** Eleonora Ficiarà, Ilaria Stura, Caterina Guiot

**Affiliations:** Department of Neurosciences, University of Turin, 10124 Turin, Italy

**Keywords:** iron, aging, neurodegeneration, Alzheimer’s disease

## Abstract

The alteration of iron homeostasis related to the aging process is responsible for increased iron levels, potentially leading to oxidative cellular damage. Iron is modulated in the Central Nervous System in a very sensitive manner and an abnormal accumulation of iron in the brain has been proposed as a biomarker of neurodegeneration. However, contrasting results have been presented regarding brain iron accumulation and the potential link with other factors during aging and neurodegeneration. Such uncertainties partly depend on the fact that different techniques can be used to estimate the distribution of iron in the brain, e.g., indirect (e.g., MRI) or direct (post-mortem estimation) approaches. Furthermore, recent evidence suggests that the propensity of brain cells to accumulate excessive iron as a function of aging largely depends on their anatomical location. This review aims to collect the available data on the association between iron concentration in the brain and aging, shedding light on potential mechanisms that may be helpful in the detection of physiological neurodegeneration processes and neurodegenerative diseases such as Alzheimer’s disease.

## 1. Introduction

Iron is distributed and appropriately balanced in the human body to perform its necessary metabolic activity [1].

Of the 4–5 g of iron in our body, most of it (65%) is bound in red blood cell haemoglobin (Hb), while 30–35% is stored in the liver in the form of ferritin, acting as an essential micronutrient in the processes of erythropoiesis, oxidative metabolism, and cellular immune responses [2].

The brain is among the most metabolically active organs in the body and accounts for at least 20% of the body’s energy consumption, although representing only about 2% of its weight. About 75–80% of the energy supports neuronal activity (axonal and synaptic signaling, but the major part is used post-synaptically [3]), with the remainder utilised to sustain the functions of astrocytes, oligodendrocytes, and microglia [4].

It acts also as co-factor in oxygen transportation, DNA synthesis, mitochondrial respiration, myelin synthesis, neurotransmitter synthesis, and metabolism [5,6], but can become neurotoxic when there is excessive intracellular accumulation [7,8].

During the lifespan, the human brain undergoes several microstructural changes, including grey matter atrophy, myelin degeneration, and iron accumulation. The alteration of iron homeostasis, in particular, can lead to neurotoxicity, driving physiological pathways towards pathological situations.

Abnormal iron metabolism is common in most neurological diseases. The pathology most directly related to iron accumulation is neurodegeneration with brain iron accumulation (NBIA), a group of inherited neurologic disorders in which iron accumulates in the basal ganglia, resulting in progressive dystonia, spasticity, parkinsonism, neuropsychiatric abnormalities, and optic atrophy or retinal degeneration [9].

Iron accumulation is a recurring pathological phenomenon in many neurological diseases including Parkinson’s disease, Alzheimer’s disease (AD), and amyotrophic lateral sclerosis [10].

Iron overload and ferroptosis are highly involved in the pathophysiological process of traumatic brain injury [11] and following acute ischaemic stroke, where the levels of hepcidin and soluble hemojuvelin (HJV) in blood are increased [12,13].

Cerebral accumulation of non-haem iron occurs heterogeneously in the brain environment during normal aging, and increased subcortical iron is associated with decreased cognitive and motor functions in the elderly population [14,15].

Although several alterations in cerebral iron metabolism could be investigated, in this research we focused on the quantitative evaluation of the iron content in the brain.

The objective of this review is to present the state-of-the art of the available evidence regarding iron load in the brain at different ages, investigating both the quantitative measurements performed with different techniques in different brain regions and also the potential accumulation mechanisms involved in normal aging and neurodegenerative processes.

## 2. Determination of Iron Deposition in Brain

Quantitative measures of iron deposition are normally performed on post-mortem specimens (which are hopefully less frequently available for young people).

Different techniques, either direct or indirect, can be used to estimate the distribution of iron in the brain regions.

Iron can be present in different forms, e.g., unbound/ bound to proteins, ferrous (Fe^2+^) or ferric (Fe^3+^). When not specified, the amount of total iron is considered.

Some direct data, e.g., [16], were obtained using graphite furnace atomic absorption spectrometry after microwave-assisted acid digestion of the samples in post-mortem excisions from 14 different areas of human brains (frontal cortex, superior and middle temporal, caudate nucleus, putamen, globus pallidus, cingulated gyrus, hippocampus, inferior parietal lobule, visual cortex of the occipital lobe, midbrain, pons (locus coeruleus), medulla, and cerebellum (dentate nucleus)) from 42 healthy adults (71 ± 12 years old, range: 53–101 years old). It was found that the iron distribution in the adult human brain was quite heterogeneous, with it being found in the largest amounts in the putamen and the lowest amounts in the medulla. Additionally, a positive correlation was found between iron levels and age in the basal ganglia (caudate nucleus, putamen, and globus pallidus).

A significant concentration of iron in the putamen was also noted in [17] after being measured by inductively coupled plasma-mass spectrometry (ICP-MS). Using the Allen Human Brain Atlas [18], iron levels were determined to be related to the transcriptomic expression of iron regulatory proteins, with researchers finding that approximately 60% of iron was associated with ferritin, equating to approximately 25% of total tissue iron essentially in storage.

Recently, in [19], correlative microscopy and spectroscopy were used to investigate the abnormal accumulation of metal traces as well as to evaluate their valence, with it being found that Fe^2+^, which is expected to produce free radicals, inducing oxidative damage and eventually causing AD, were actually present in all the deposits in the brain tissue of three AD patients.

Indirect evaluations, mainly based on brain magnetic resonance imaging (MRI), are much more diffused.

Some quantitative MRI methods are sensitive to brain iron, such as transverse relaxation rate R2* (1/T2*) and quantitative susceptibility mapping (QSM), which indicate to what extent the ‘biological material’ may be magnetised in an external magnetic field. Both techniques demonstrate a strong correlation with post-mortem iron measures and have highly variable concentrations in specific anatomical structures [20,21].

The method based on the calculation of the relaxation time constant (1/T2* = 1/T2 + 1/T2′) accounts for local inhomogeneities in the brain tissue, to which the presence of iron contributes. Furthermore, as for R2, R2* [22] requires multi-echo scans that are prone to subject motion and other errors [23]. To investigate the magnetic properties of local tissues, their magnetic susceptibility can be quantified by QSM [24]. QSM is used not only to study iron distribution, but also to investigate metabolic oxygen consumption, blood degradation, calcification, demyelination, and other pathophysiological susceptibility changes, as well as contrast agent biodistribution in MRI [24]. In fact, the magnetic susceptibility of cerebral tissue is affected by myelin, calcium, and deoxyhaemoglobin. However, in the basal ganglia and other grey matter structures, QSM is mainly influenced by ferritin iron saturation [25].

Both of these MRI techniques enable in vivo studies and offer the opportunity to correlate regional iron concentrations in brain tissue to clinical phenotypes [14]. Moreover, R2* and QSM presumably provide complementary information, given that both increase along with iron, while myelin elevates R2* but decreases QSM [26].

Many MRI-estimated parameters have been validated by comparison with post-mortem iron concentration data—e.g., [27] detected T*-weighted spin-echo sequences from the globus pallidus and putamen, [28] evaluated the magnetic susceptibility of four iron-rich brain regions (putamen, globus pallidus, caudate, and thalamus), [29] combined T2* relaxometry and automatic segmentation from some subcortical structures to estimate brain volume and iron deposition, and [21] developed a QSM which was strongly correlated to the chemically detected iron concentration in deep grey matter (less so in white matter).

A different technique was proposed by [30], based on magnetoencephalogram showing that the accumulation of magnetite in the hippocampus and nearby regions in old men could be satisfactorily detected.

The iron detected by MRI cannot in principle be attributed only to non-haem but can derive from silent microhaemorrhages—e.g., in [31] haem-rich deposits in the cortex were detected by histochemical exams, especially in older patients exhibiting senile plaques. Furthermore, brain haemosiderin deposits of ischaemic origin were detected by MRI in the putamen of 200 patients [32].

Finally, the choroid plexuses also serve as iron storage tissue, and MRI susceptibility-weighted imaging (SWI) was used to evaluate iron accumulation in the choroid plexus following transfusion in patients with beta-thalassemia major [33].

## 3. Iron Brain in Aging

Based on such indirect techniques, many investigations of iron concentration and aging have been conducted, focusing on different brain regions, especially on the so-called ‘basal ganglia’ and some parts of the cortex related to motor control.

**Figure 1:**
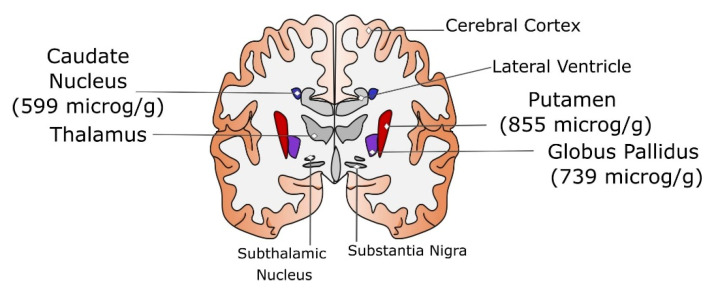
Sketch of the coronal section of the brain where several structures belonging to basal ganglia are detailed. For putamen, globus pallidus, and caudate nucleus, the mean iron levels (in μg/g) reported by Ramos et al. [16] are given. The regions with the smallest value of deposited iron are filled in in blue, in red the highest ones are shown, and in violet the intermediate scores are given. The structures not analysed in detail are filled in in grey.

In Figure 1, a sketch of the brain anatomy is given together with the normal iron content of the specific regions analysed below.

In [34], the frontal white matter, caudate, putamen, and globus pallidus of 13 adult males were examined, with a significant age-related increase in the iron-related MRI parameter being found. The relationship between age and amount of basal ganglia iron in 20 normal individuals ranging from 24 to 79 years of age can be found with an in vivo MRI method, enabling the determination of a quantitative index of local brain iron, showed a strong direct relationship between age and regional iron content in the putamen and caudate but not in the globus pallidus or thalamus [35].

Similarly, in 11 young and 12 elderly healthy adults, the amount of iron was found to be high in the globus pallidus regardless of age and significantly increased in the putamen with advancing age [36]. By using quantitative magnetic resonance parameters sensitive to complementary tissue characteristics (volume atrophy, iron deposition, and microstructural damage) in 100 healthy subjects, large age-related variations were observed in the thalamus, putamen, and caudate but not in the hippocampus, amygdala, pallidum, or accumbens [37]. Additionally, other studies conducted on large cohorts of healthy adults such as [23] have confirmed that brain cells accumulate iron as a function of aging depending on their anatomical location, and that the subcortical regions and the frontal lobe are involved. Since then, the cortex has also been extensively studied.

A longitudinal study conducted over 7 years based on T1 relaxometry showed a T1 decrease in the lateral frontal, parietal, and temporal cortex, while mean white matter T1 values remained stable [38]. In [39], it was shown that the iron accumulation in the basal ganglia increased linearly with age, whereas in the thalamus, pulvinar, precentral cortex, and precuneus, it followed a quadratic or exponential pattern. In the cortex, the accumulation of iron increases with age mainly in the areas related to motor, cognitive, and visual functions. Four primary deep grey matter regions (thalamus, putamen, caudate, and globus pallidus) in 498 healthy individuals aged 5–90 years were studied [26], showing that in the caudate, putamen, and globus pallidus, the increases in iron-related MRI parameters were steepest during childhood and continued gradually throughout adulthood, except for caudate susceptibility, which reached a plateau in the late 30s, and in the thalamus, which reached a plateau in the mid-30s to early 40s and decreased thereafter.

Iron content at different ages has also been studied in connection with brain efficiency. For instance, [40] assessed iron deposits in the basal ganglia and microbleeds as well as cognitive ability in 143 healthy subjects. They found that possessing more iron deposits at age 72 was significantly associated with lower general cognitive ability at ages 11, 70, and 72, suggesting that iron deposits are related to lifetime cognitive decline. A total of 113 healthy adults (age 19–83 years) were investigated to determine the brain volume and iron concentration in the hippocampus, caudate nucleus, and primary visual cortex by assessing their memory performance [41], with the researchers finding a significant association between age-related decline in memory, reduced brain volume, and increase in free iron concentration only in the hippocampus. On the contrary, upon comparing middle-aged and older patients, the highest iron concentration in the globus pallidus and pallidal and putaminal iron were found to be significantly and inversely associated with cognitive performance in all cognitive domains except for memory [14]. It is unclear if age-related increases in hippocampus iron concentration reflect a normal aging process or represent an early physiological sign of impending pathological decline [41].

Being implicated in dementia and memory loss, the hippocampus has been intensively studied. Iron deficiency in the foetal and newborn periods has been related to abnormal cognitive performances and emotional regulation [42].

During childhood, age was found to correlate positively with iron content in the hippocampus and across subregions of the basal ganglia, where the iron content was always larger [43].

On a lifelong basis, an age-related increase in iron content (investigated with R2* and diffusion) was found to correlate with decreases in memory performance, but the difference in iron content was always low–moderate in the hippocampus and caudate and moderate–high in the putamen and globus pallidus. Furthermore, hippocampal restricted diffusion was found to be significantly related to memory performance [44].

The study of Acosta-Cabronero and colleagues indicated that there is a predilection for iron deposits in the frontal lobes which, when combined with subcortical findings, suggests that iron accumulation with age predominantly affects brain regions related to motor/output functions [23].

T2*-based estimates of regional iron concentration revealed significant age-related differences in multiple regions [37,45,46]. Furthermore, several studies have investigated the relation between iron (from indirect MRI parameters) and age by means of different statistical analyses (i.e., linear correlation, best fit function, mediation analysis). A detailed report is given in the Supplementary Materials (Table S1).

### Iron vs. Age Relationship: Quantitative Correlations

As previously reported, the relation between brain iron content and age is very heterogenous in different brain regions, depending on the technique used to quantify iron and the variability of the populations under study.

However, some papers have reported a quantitative description of the relation between brain iron deposition (measured in vivo by QSM) and age, allowing a comparison with the data reported from the post-mortem analysis of brain samples in the putamen, caudate nucleus, and globus pallidus (for the other regions, the equation parameters were not available).

In particular, the studies of Treit et al. [26] and Acosta-Cabronero et al. [23] report the equations for the curves of iron estimation using QSM, while for post-mortem analysis the interpolation is available from Ramos et al. [16]. Due to our interest in the aging process, we considered only the age range of 60–80 years, based on which we calculated the correlation coefficient (Pearson coefficient).

**Figure 2:**
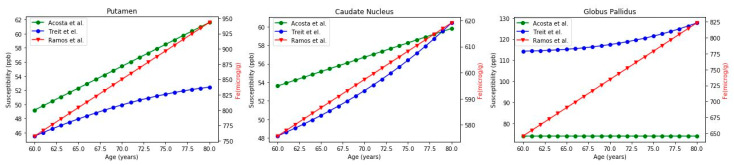
Comparison of the iron concentration estimated with QSM (susceptibility expressed in ppb, reported on the left-side scale, and interpolated by the green and blue curves) and post-mortem analysis (expressed in μg/g, reported on the right-side scale, and interpolated by the red curve) in three brain areas (putamen, caudate nucleus, and globus pallidus). On the x-axis, the age, in the range of 60–80 years, is reported [16,23,26].

The results of the analysis performed on three brain regions (putamen, caudate nucleus, and globus pallidus) are reported in Figure 2. Unfortunately, the corresponding equations for regions with not-growing trends (e.g., thalamus) were not available.

In Figure 2, the two QSM estimations (green and blue curves) are reported considering the left-side scale in ppb, while the post-mortem one (red curve) gives the right-side scale in μg/g. In Table 1, the mean iron concentration with SD, the correlation coefficients, the proportion of QSM, and post-mortem values in the three regions are reported. Even though they may seem different, a good correlation was found between the curves.

The most controversial results were related to the globus pallidus. The increase in iron with age in this region was very clear in the study of Ramos and colleagues’, much weaker in the study by Treit et al., and no variations were detected by Acosta-Cabronero et al. These conflicting results are probably due both to the different techniques used for investigation and to the deep position of the area in the brain, which could make measurements difficult.

On average, the scale factor between the values of iron obtained from post-mortem analyses (iron concentration μg/g) and susceptibility (ppb) was 11.7 ± 4.0. This could give an idea of the real value (in μg/g) of iron given QSM estimation. However, the scale factor could change significantly between regions, and more investigations in this direction should be performed.

## 4. Where Does Brain Iron Excess Come from?

### 4.1. The Main Players: Transporters, Free Iron, and the Role of the Barriers

Brain iron content increases with age could be caused by changes in various proteins that regulate the iron content.

The trafficking of iron in the brain milieu is mainly managed by the brain barrier system, with proteins, receptors, and transporters being responsible for the exchange of iron between the blood and the brain compartments [47]. The systemic organs and the brain share the same iron regulatory mechanisms and pathways based on iron-modulating proteins, providing a link to the maintenance of iron homeostasis within the brain [48].

The uptake of iron from the periphery starts mainly with the transferrin–receptor pathway in the brain endothelium through brain microvascular endothelial cells [49].

According to [50], the maintenance of iron homeostasis in the brain is the responsibility of neuroglia and possibly of the choroid plexus. The amount of transferrin, the iron transporter protein also involved in travel across the blood–brain barrier (BBB), depends on the presence of a mature population of oligodendrocytes.

Both ferritin, the iron storage protein, and free iron are found in the brain in oligodendrocytes and microglia. Additional cells in which iron and ferritin are found are tanycytes, which are associated with the third ventricle, suggesting the possible transport of iron from the cerebrospinal fluid (CSF) into the brain.

Additionally, neurons contain granular iron deposits, which are more visible with age, and ferritin and iron are present in microglial cells in all brain regions, especially in the hippocampus. In conclusion, this study indicated the existence of an important role for neuroglia in the regulation of iron in the brain, the existence of a transport system for the transfer of iron between the brain and the CSF, and an increase in stainable iron in neurons without a concomitant increase in neuronal ferritin immunostaining, suggesting that a ferritin-independent accumulation of neuronal iron with age takes place [51].

Following autopsy of three groups of patients (<65 years, >65 years, and AD), transferrin was found to be more abundant in white than in grey matter, while ferritin (around 10 times more abundant than transferrin) and iron were determined to be equally distributed in grey and white matter. AD patients had lower transferrin levels in white matter [52].

A gender dependence of brain ferritin iron, which is present in lower amounts in women in the caudate, thalamus, frontal lobe, and corpus callosus, was detected by [53].

Additionally, hepcidin and ferroportin (Fpn), which are important iron-regulatory proteins, may be implicated, since their presence in neurons and astrocytes is reduced in mouse AD models, where the reduction in Fpn levels is associated with cerebral ischaemia, inflammation, neuronal loss, senile plaque formation, and possibly the aging process itself. A possible reduction in the passage of hepcidin across damaged vascular endothelium has been hypothesised [54]. A contribution to ferroptosis derives from the reduction in Fpn, as observed following the genetic deletion of Fpn in principal neurons of the neocortex and hippocampus by breeding Fpn fl/fl mice with NEX-Cre mice [55], which also showed significant memory impairment.

Moreover, HJV plays a particularly important role in this process. HJV undergoes complicated post-translational processing in an iron-dependent manner and interacts with multiple proteins that are essential for iron homeostasis, such as hepcidin [56].

Furthermore, Divalent Metal Transporter 1 (DMT1), which mediates the intracellular transport of iron and is responsible for non-transferrin bound iron (NTBI) transport, regulation, and compartmentalisation in specific brain regions plays important roles during aging.

Other details on several iron-related proteins detected in biofluids can be found in [47].

### 4.2. Blood–Brain Barrier and Age

The BBB is considered the main route by which iron travels from the blood to the brain. The BBB acts as a barrier that selects what can enter the brain and is formed by tight junctions between endothelial cells lining blood vessels, the end-feet of astrocytes, and a basement membrane.

Interestingly, the BBB, which is vital for maintaining brain homeostasis, breaks down with age and further disruption is a hallmark of many age-related disorders [57]. The alterations and breakdown of functional components of the BBB can occur naturally with aging even in the absence of underlying conditions that cause cognitive decline and dementia [58].

Moreover, the systems regulating the circulation of brain extracellular fluid and CSF, such as the glymphatic system, are also less efficient with healthy aging [59].

There is growing interest in new methodologies for quantifying the spatial organisation and the intersubject variability of cerebral vessels. Non-invasive imaging techniques, such as time-of-flight magnetic resonance angiography (ToF-MRA) and SWI, also referred to as high-resolution venous venography, can visualise the large arteries and veins, respectively, on a single-subject basis [60]. It has been shown that cerebral vasculature is highly heterogeneous, displaying disproportionally large vessel densities in brain areas such as the anterior and posterior cingulate, cuneus, precuneus, parahippocampus, insula, and temporal gyri [60].

BBB permeability changes in healthy and diseased states are difficult to measure, but dynamic contrast-enhanced magnetic resonance imaging (DCE-MRI) can be used to estimate the influx constant Ki based on the movement of gadolinium contrast into the brain [61]. Higher Ki values in the grey matter of healthy individuals versus their white matter and in multiple sclerosis (MS) lesions versus normal-appearing white matter were measured.

### 4.3. The Interplay of Iron with Other Factors

#### 4.3.1. Potential Iron Alteration Due to Microbleeds

Interestingly, the onset of microbleeds may be a factor that should be taken into account in alterations of iron concentration. In the presence of silent microbleeds, haem-related iron can travel across barriers. Haem is degraded by the stress protein Haem Oxygenase-1 (HO-1) to biliverdin/bilirubin, CO, and free iron. According to [62], HO-1 over-expression contributes to the pathological iron deposition and mitochondrial damage documented in aging-related neurodegenerative disorders. In the normal Central Nervous System (CNS), HO-1 mRNA and protein are normally confined in scattered neurons and neuroglia, but the HO-1 gene is upregulated in glial cells within MS plaques in the vicinity of human cerebral infarcts, haemorrhages, and contusions, as well as in various other degenerative and non-degenerative human CNS disorders. Moreover, HO-1 hyperactivity promotes the mitochondrial sequestration of NTBI in oxidatively challenged astroglia and may thereby contribute to pathological iron deposition [63].

MRI sequences such as SWI enable the simultaneous quantitation of regional brain iron levels and brain microbleeds (BMBs) serially, which is useful in the study of dementia [64]. Interestingly, regional iron changes and the presence of BMBs can be considered MRI-based biomarkers for neurodegenerative diseases [64].

Furthermore, alternative interpretations of MRI T2* shortening in the hippocampus should be considered. It is possible that T2* decreases with age due to changes in the brain vasculature [41]. Differences in T2* may stem from alterations in cerebral microvascular dynamics and tissue oxygenation or from changes in non-haem iron homeostasis [41]. The age-related shortening of T2* could also reflect hemosiderin in cerebral microbleeds. Although BMBs are relatively common among the general elderly population [65], they are associated with less-than-optimal cognitive status [66].

In [14], the highest iron concentration was found in the globus pallidus; pallidal and putaminal iron was significantly and inversely associated with cognitive performance in all cognitive domains, except for memory. These associations were iron-load-dependent. Vascular brain lesions and brain volume did not mediate the relationship between iron and cognitive performance, indicating that higher R2*-determined iron in the basal ganglia correlates with cognitive impairment during brain aging, independent of concomitant brain abnormalities. The prognostic significance of this finding needs to be further investigated [14].

#### 4.3.2. Interaction of Iron with Amyloid Beta-APP and Tau

The relationship between iron and amyloid beta (Aβ) protein has been investigated for a long time. In 1999 [67], the stabilisation of the more damaging ferrous (Fe^2+^) to Fe^3+^ in physiological conditions was confirmed to be significantly slowed in the presence of aluminium salts as well as in the presence of Aβ fragments.

Early plaque formation was found to be related to elevated iron content [68] in the cortex and hippocampus in a PSAPP mouse model. Until Aβ plaques reach a certain level, neither elevated NTBI uptake nor iron toxicity in hippocampal neurons were observed [69].

The interactions between Aβ and haemoglobin (Hb) which possibly induce plaque formation were also investigated in prior research [70], showing that Hb binds to Aβ and co-localises with the plaque and vascular amyloid deposits in post-mortem AD brains, as well as that the formation of an envelope-like structure composed of Aβ surrounding the Hb droplets can be observed after the microinjection of human Hb into the dorsal hippocampi of APP/PS1 transgenic mice. More chemical details on the interaction of haem and Aβ peptides are given in [71].

After realising that Aβ is diamagnetic and can generate strong contrast in susceptibility maps, experiments were performed on transgenic mouse models over a period of 18 months. In these, QSM was used to longitudinally monitor Aβ accumulation and accompanying iron deposition in vivo [72], confirming their interplay.

Additionally, Tau aggregation was shown to be related to cortical iron accumulation using MRI and tau-PET [73].

More generally, the formation of neuritic plaques is strictly related to the droplet degeneration of hippocampal and cortical neurons [74], consisting of their breaking up into spheres of p-tau droplets of various sizes which are encased in deposits. This occurs as a consequence of a massive upregulation of ferritin in the microglia (detected by Perl’s Prussian blue), suggesting the presence of high levels of free iron.

To conclude, the most convincing view is that the presence of free iron elicits the formation of enveloping plaques, reducing its toxicity in the same manner as the oyster surrounding a grain of sand, forming a pearl. Contrasting iron accumulation (e.g., with chelating therapies) would prevent plaque formation and the following neurodegenerative effects.

#### 4.3.3. ApoE and the Genetic Origin of AD

Metals—iron in particular—also interact with other proteins, including apolipoprotein E (ApoE) [75].

An unexpected link between ApoE and brain iron was recently shown [76], with ApoE being shown to act as a potent inhibitor of ferroptosis (EC50 ≈ 10 nM; N27 neurons) by activating the PI3K/AKT pathway, which inhibits the autophagic degradation of ferritin (ferritinophagy).

This result could be related to the well-known association of the ApoE gene with AD, with ApoE4 representing the strongest genetic risk factor for the development of late-onset AD. Additionally, ferritin levels in the CSF are increased in patients with the ApoE4 allele, and increased CSF ferritin levels are associated with earlier disease onset [77].

A multivariate genomic scan reported that ApoE, haem-regulation, and plasma iron levels were the strongest factors associated with human health span and lifespan [78]. Iron was also shown to impact AD as a major complication of aging [79].

It was recently observed that ApoE secretion is regulated by presenilin (PS) [80], is completely abolished in PS-deficient cells, and markedly decreased by the inhibition of gamma-secretase activity.

Both PS and ApoE4 genes could therefore influence the ability of ApoE to inhibit ferroptosis and its chain of consequence in the brain.

This could also shed some light on early or familial AD related to mutations in PS and APP.

We can hypothesise that the ferroptosis mechanisms already induced by low levels of iron accumulation (occurring in young brains) cannot be counteracted by ApoE secretion, which is reduced by genetic factors.

**Figure 3:**
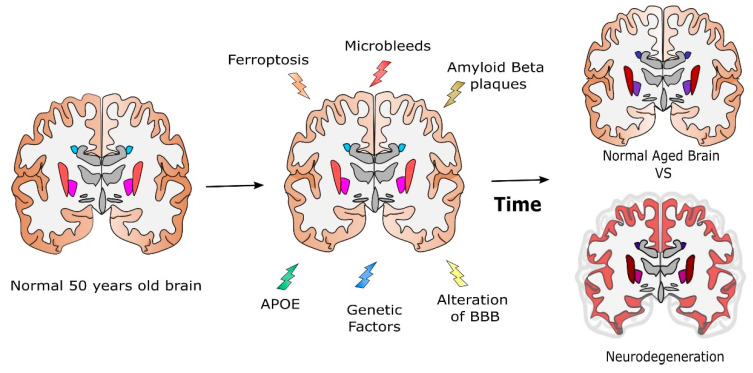
Potential mechanisms involved in the modulation of the brain iron concentration during aging compared with those leading to neurodegeneration and AD. Differences in brain areas are already present in normal brains, but genetic factors, ferroptosis, microbleeds, and other alterations can shift the iron content values from what is expected in a normal aged brain to larger values (darker colours), predicting neurodegeneration.

All the previous hypotheses have been collected and are represented in Figure 3.

## 5. Discussion and Conclusions

In this work, we collected, to the best of our knowledge, the available data on the brain iron content throughout the lifespan, especially at advanced ages.

Our investigation highlights the relation between brain iron concentration and aging for several brain regions (in particular, those belonging to the basal ganglia), giving an estimate of the relationship between the indirect values of iron concentration (by QMS in vivo) and those directly obtained from post-mortem samples.

The different scale factors given for the QSM measurements may be due to the different experimental procedures used and the variability of the samples.

However, QSM reliably quantifies changes in iron content in the deep grey matter structures, and the accuracy of QSM in identifying iron deposition in these regions has been validated in post-mortem studies, showing significant correlations between QSM contrast and the histochemical measurement of iron [21,81,82,83]. Furthermore, the increased magnetic susceptibility in the basal ganglia most likely arises from an increase in iron content (except when other paramagnetic metals are involved), while QSM increases in the white matter can be the result of an increase in iron, a decrease in myelin (demyelination), or both [82].

The systematic review of Ravanfar and colleagues [84] provides a synthesis of the findings from existing QSM studies in neurodegenerative disease. As a general pattern, QSM revealed increased magnetic susceptibility (suggestive of increased iron content) in the brain regions associated with the pathology of several disorders, such as AD and Parkinson’s disease [84].

Due to its high metabolic requirements, the brain is supplied by a dense arterial network [85]. The anatomical structures where vessels are particularly prominent either because of their size or their tendency to cluster were investigated in [86].

Variations in the iron concentration patterns during aging among the different subcortical regions could possibly be related to the variable distribution of vascular networks, as well as the differential deterioration of the BBB structure during aging across these regions. Consequently, iron could be delivered differentially to the tissues.

Some anatomical structures are affected by the presence of vascular structures deeply embedded in the brain parenchyma and hence tightly coupled with tissue. At this other extreme of the range, co-localisation was not found to depend on the misregistration of the parenchyma and the spaces containing the vascular tree. The basal ganglia and especially the ventral striatum are examples of this close relationship between vessels and tissue [86].

Several iron-related proteins and transporters regulate the iron passage across the brain barriers necessary for the brain iron cycle.

For example, DMT1 mediates the crosstalk between CNS and peripheral tissues by systemic diffusion through the BBB, with the involvement of peripheral iron homeostasis in association with inflammation, showing a complex pattern of interrelationship of iron levels with aging, neurodegeneration, and neuroinflammation [87].

DMT1 was also studied in cerebral haemorrhage models undergoing iron chelation by deferoxamine in order to examine the mechanism of iron-induced BBB disruption after subarachnoid haemorrhage and investigate the potential therapeutic effect of iron chelation on subarachnoid haemorrhage [88].

Interestingly, the prevalence of cerebral microbleeds and intracerebral haemorrhage (and consequently the presence of haem and iron in the brain parenchyma) increases with aging, and there is evidence that cerebral microbleeds are more prevalent in patients with AD than in the general population [89].

Furthermore, haem-rich deposits are a common feature of the aging cortex and these deposits represent sites of intracerebral bleeding [31]. Another study indicated that senile plaques are sites of microhaemorrhages, suggesting that microhaemorrhages can be early events in plaque formation [90].

In conclusion, the lifespan accumulation of iron in several brain regions can be considered a multifactorial process, involving damage to BBB and interaction with other factors, such as the presence of Aβ plaques and mechanisms involving ApoE.

Differences in brain areas are already present in normal brains, but genetic factors, ferroptosis, the presence of microbleeds, and other alterations due to plaque accumulation could increase the iron content values from what would be expected in the normal aged brain to larger values.

All these elements may contribute to different extents to healthy aging, moderate physiological neurodegeneration, or even to the onset of neurodegenerative diseases such as AD.

The data collected in this paper may be very useful for designing quantitative models of physiological and pathological iron exchange in the brain [91,92].

## Figures and Tables

**Table 1:** Mean iron concentration estimated from QSM and post-mortem analyses in putamen, caudate nucleus, and globus pallidus in the three considered papers. The correlation coefficient (Pearson, r) was calculated between susceptibility and values obtained from post-mortem analysis. The proportion was calculated by dividing post-mortem values by QSM values.

Brain Region	Ref	Mean Iron Concentration (60–80 Years)	Correlation Coefficient	Proportion
Putamen	Treit et al. [26] Acosta et al. [23] Ramos et al. [16]	59.15 ± 5.34 ppb 55.40 ± 3.75 ppb 850.37 ± 56.17 μg/g	r = 0.99, *p* = 2.6 × 10^−17^ 1	(17.1 ± 0.4) (15.4 ± 0.0)
Caudate Nucleus	Treit et al. [26] Acosta et al. [23] Ramos et al. [16]	53.54 ± 3.67 ppb 56.7 ± 1.88 ppb 597.37 ± 13.14 μg/g	r= 0.99, *p* = 6.8 × 10^−20^ 0.99	(11.2 ± 0.5) (10.5 ± 0.1)
Globus Pallidus	Treit et al. [26] Acosta et al. [23] Ramos et al. [16]	118.72 ± 4.11 ppb 74 ppb 734.42 ± 53.90 μg/g	r= 0.96, *p* = 9.8 × 10^−12^	(6.2 ± 0.3) (9.9± 0.7)

## Data Availability

Data are available upon request to the corresponding author.

## Supplementary Materials

The following supporting information can be downloaded at: https://www.mdpi.com/article/10.3390/ijms231710018/s1. References [93,94] are cited in Supplementary Materials.

## Abbreviations

Magnetic Resonance Imaging	MRI
Transverse Relaxation Rate	R2* (1/T2*)
Quantitative Susceptibility Mapping	QSM
Susceptibility Weighted Imaging	SWI
Non-Transferrin Bound Iron	NTBI
Cerebro Spinal Fluid	CSF
Blood–brain barrier	BBB
Brain Microbleeds	BMB
Ferroportin	Fpn
Amyloid Beta Protein	Aβ
Amyloid Precursor Protein	APP
Pre Senilin	PS
Apolipoprotein E	ApoE
Hemojuvelin	HJV
