# Supplementary material for: Iron Deposition in Brain: Does Aging Matter?
Source: Int J Mol Sci. 2022 Sep 2;23(17):10018. doi: 10.3390/ijms231710018 (PMC9456423; doi:10.3390/ijms231710018)
Supplement: Supplementary file 1 [file ijms-23-10018-s001.zip › ijms-1865023-supplementary.pdf]

Supplementary Materials

Table S1 collected information about iron concentration and age in healthy people in different brain regions: Whole brain, Cortex, White matter, Putamen, Substantia Nigra, Red Nucleus, Thalamus, Globus Pallidus, Caudate Nucleus, Amygdala, Hippocampus, Dentate Nucleus. For each brain regions, we indicated the following details:

- Technique used for iron estimation (i.e., post-mortem analysis, in vivo MRI) and the relative parameters (i.e., T1, R2*)
- Number of patients and age range (years) of the population
- Quantitative information for the relation between estimated iron concentration and age (i.e., Pearson coefficient, interpolation, regression coefficient)

**Brain region**	**Ref**	**Technique for iron estimation**	**N° of patients (N° of females)**	**Age range**	**Relation Iron vs Age**
Whole	[1]	Post-mortem	42 dead (F=15)	53-101 years	r^2=0.1534 (NS)
Cortex	[2]	T1	17 healthy (F=8)	51-77 years	decreasing T1 rate in time= 5.1 ± 7.81 ms/year ($p = 0.039$)<br>$r = -0.59$ ($p = 0.012$) relationship T1/iron

White matter	[2]	T1	17 healthy (F=8)	51-77 years	decreasing T1 rate in time p = 0.38
	[3]	R2*	140 healthy (F=81)	20-74 years	r^2=0.37
	[4]	T1, QSM	95 healthy (F=57)	21-58 years	r^2=0.09
Putamen	[5]	QSM	67 healthy (F=35)	18-78 years	r=0.3780 (Pearson) (p=0.001)
	[6]	R2*, QSM	498 healthy (F=286)	5-90 years	R2*(s-1)=0.58*Age-0.0008*Age^2+0.00004*Age^3+13.2<br>Susceptibility(ppb)=0.88*Age+0.002*Age^2+0.000055*Age^3-2.6
	[7]	Post mortem	7 dead (F=4)	57-91 years	r^2=0.17 (p=0.47)

	[1]	Post mortem	42 dead (F=15)	53-101 years; 5 age groups	r^2=0.151 (NS)
	[3]	R2*	140 healthy (F=81)	20-74 years	r^2>0.4
	[8]	QSM	116 healthy (F=56)	20-79 years	ρ^2=0.48 (p=6*10^-18)
	[4]	T1, QSM	95 healthy (F=57)	21-58 years	r^2=0.39 (p<0.001)
	[9]	R2*	336 healthy (F=204)	38-86 years	Regression coefficient (beta= 2.55, 95% CI 1.12, 3.97, p =0.01)
	[10]	T2*, R2*	100 healthy (F=55)	20-70 years	beta=0.466 (p<0.001)
Substantia Nigra	[5]	QSM	67 healthy (F=35)	18-78 years	r=0.0437 (NS)

	[8]	QSM	116 healthy (F=56)	20-79 years	ρ^2=0.21 (p=3*10^-7)
	[4]	T1, QSM	95 healthy (F=57)	21-58 years	r^2=0.17 (p<0.001)
Red Nucleus	[5]	QSM	67 healthy (F=35)	18-78 years	r=0.4840 (p<0.0001)
	[8]	QSM	116 healthy (F=56)	20-79 years	ρ^2=0.32 (p=3*10^-11)
	[4]	T1, QSM	95 healthy (F=57)	21-58 years	r^2=0.32 (p<0.001)
Thalamus	[5]	QSM	67 healthy (F=35)	18-78 years	r=0.0784 (NS)
	[8]	QSM	116 healthy (F=56)	20-79 years	ρ^2=0.05 (NS)
	[7]	Post mortem	7 dead (F=4)	57-91 years	r^2=0.14 (p=0.47)

	[6]	R2*, QSM	498 healthy (F=286)	5-90 years	R2*(s-1)=1.17*Age-0.0008*Age^2+0.00004*Age^3+13.2<br>Susceptibility(ppb)=0.88*Age+0.002*Age^2+0.000055*Age^3-2.6
	[4]	T1, QSM	95 healthy (F=57)	21-58 years	r^2=0.06 (NS)
	[10]	T2*, R2*	100 healthy (F=55)	20-70 years	beta=0.136 (p=0.050)
Globus Pallidus	[5]	QSM	67 healthy (F=35)	18-78 years	r=0.3186 (NS)
	[8]	QSM	116 healthy (F=56)	20-79 years	ρ^2=0.01<br>(NS)

	[6]	R2*, QSM	498 healthy (F=286)	5-90 years	R2*(s-1)=0.32*Age-0.02*Age^2+0.00013*Age^3+18.1<br><br>Susceptibility(ppb)=4.39*Age-0.09*Age^2+0.0006*Age^3-45.3
	[1]	Post mortem	42 dead (F=15)	53-101 years; 5 age groups	r^2=0.078 (NS)
	[4]	T1, QSM	95 healthy (F=57)	21-58 years	r^2=0.16 (p=0.22)
	[10]	T2*, R2*	100 healthy (F=55)	20-70 years	beta=0.281 (p=0.005)
Caudate Nucleus	[5]	QSM	67 healthy (F=35)	18-78 years	r=0.0474 (NS)
	[8]	QSM	116 healthy (F=56)	20-79 years	ρ^2=0.23<br><br>(p=7*10^-8)

	[6]	R2*, QSM	498 healthy (F=286)	5-90 years	R2*(s-1)=0.43*Age-0.007*Age^2+0.00004*Age^3+13.7<br><br>Susceptibility(ppb)=1.85*Age-0.03*Age^2+0.0002*Age^3 +2
	[11]	T2*	113 Healthy (F=76)	19-83 years	−0.54 ($p<0.05$)
	[1]	Post mortem	42 dead (F=15)	53-101 years; 5 age groups	$r^2=0.0094$ (NS)
	[3]	R2*	140 healthy (F=81)	20-74 years	$r^2>0.4$
	[4]	T1, QSM	95 healthy (F=57)	21-58 years	$r^2=0.18$ ($p<0.001$)
	[10]	T2*, R2*	100 healthy (F=55)	20-70 years	beta=0.1395 ($p<0.001$)

Amygdala	[5]	QSM	67 healthy (F=35)	18-78 years	r=0.2538 (NS)
	[8]	QSM	116 healthy (F=56)	20-79 years	ρ^2=0.01 (NS)
	[10]	T2*, R2*	100 healthy (F=55)	20-70 years	beta=0.251 (p=0.015)
Hippocampus	[5]	QSM	67 healthy (F=35)	18-78 years	r=0.0300 (NS)
	[8]	QSM	116 healthy (F=56)	20-79 years	ρ^2=0.03 (NS)
	([11]	T2*	113 Healthy (F=76)	19-83 years	−0.62 (p>0.05)
	[10]	T2*, R2*	100 healthy (F=55)	20-70 years	beta=0.367 (p=0.004)
Dentate Nucleus	[5]	QSM	67 healthy (F=35)	18-78 years	r=0.2170 (NS)

	[8]	QSM	116 healthy (F=56)	20-79 years	ρ^2=0.14 (p=5*10^-5)
	[4]	T1, QSM	95 healthy (F=57)	21-58 years	r^2=0.08

F: Female; QSM: Quantitative Susceptibility Mapping

Beta: regression coefficient; r^2: Correlation Coefficient , Pearson, NS: Not Significant

Bibliography

1. Ramos, P.; Santos, A.; Pinto, N.R.; Mendes, R.; Magalhães, T.; Almeida, A. Iron Levels in the Human Brain: A Post-Mortem Study of Anatomical Region Differences and Age-Related Changes. *Journal of Trace Elements in Medicine and Biology* **2014**, *28*, 13–17, doi:10.1016/j.jtemb.2013.08.001.
2. Gracien, R.-M.; Nürnberger, L.; Hok, P.; Hof, S.-M.; Reitz, S.C.; Rüb, U.; Steinmetz, H.; Hilker-Roggendorf, R.; Klein, J.C.; Deichmann, R.; et al. Evaluation of Brain Ageing: A Quantitative Longitudinal MRI Study over 7 Years. *Eur Radiol* **2017**, *27*, 1568–1576, doi:10.1007/s00330-016-4485-1.
3. Cherubini, A.; Caligiuri, M.E.; Péran, P.; Sabatini, U.; Cosentino, C.; Amato, F. Importance of Multimodal MRI in Characterizing Brain Tissue and Its Potential Application for Individual Age Prediction. *IEEE Journal of Biomedical and Health Informatics* **2016**, *20*, 1232–1239, doi:10.1109/JBHI.2016.2559938.
4. Burgetova, R.; Dusek, P.; Burgetova, A.; Pudlac, A.; Vaneckova, M.; Horakova, D.; Krasensky, J.; Varga, Z.; Lambert, L. Age-Related Magnetic Susceptibility Changes in Deep Grey Matter and Cerebral Cortex of Normal Young and Middle-Aged Adults Depicted by Whole Brain Analysis. *Quantitative Imaging in Medicine and Surgery* **2021**, *11*, 3906919–3903919, doi:10.21037/qims-21-87.
5. Howard, C.M.; Jain, S.; Cook, A.D.; Packard, L.E.; Mullin, H.A.; Chen, N.; Liu, C.; Song, A.W.; Madden, D.J. Cortical Iron Mediates Age-Related Decline in Fluid Cognition. *Human Brain Mapping* **2022**, *43*, 1047–1060, doi:10.1002/hbm.25706.

6. Treit, S.; Naji, N.; Seres, P.; Rickard, J.; Stolz, E.; Wilman, A.H.; Beaulieu, C. R2* and Quantitative Susceptibility Mapping in Deep Gray Matter of 498 Healthy Controls from 5 to 90 Years. *Human Brain Mapping* **2021**, *42*, 4597–4610, doi:10.1002/hbm.25569.
7. McAllum, E.J.; Hare, D.J.; Volitakis, I.; McLean, C.A.; Bush, A.I.; Finkelstein, D.I.; Roberts, B.R. Regional Iron Distribution and Soluble Ferroprotein Profiles in the Healthy Human Brain. *Progress in Neurobiology* **2020**, *186*, 101744, doi:10.1016/j.pneurobio.2019.101744.
8. Acosta-Cabronero, J.; Betts, M.J.; Cardenas-Blanco, A.; Yang, S.; Nestor, P.J. In Vivo MRI Mapping of Brain Iron Deposition across the Adult Lifespan. *J. Neurosci.* **2016**, *36*, 364–374, doi:10.1523/JNEUROSCI.1907-15.2016.
9. Ghadery, C.; Pirpamer, L.; Hofer, E.; Langkammer, C.; Petrovic, K.; Loitfelder, M.; Schwingenschuh, P.; Seiler, S.; Duering, M.; Jouvent, E.; et al. R2* Mapping for Brain Iron: Associations with Cognition in Normal Aging. *Neurobiology of Aging* **2015**, *36*, 925–932, doi:10.1016/j.neurobiolaging.2014.09.013.
10. Cherubini, A.; Péran, P.; Caltagirone, C.; Sabatini, U.; Spalletta, G. Aging of Subcortical Nuclei: Microstructural, Mineralization and Atrophy Modifications Measured in Vivo Using MRI. *NeuroImage* **2009**, *48*, 29–36, doi:10.1016/j.neuroimage.2009.06.035.
11. Rodrigue, K.M.; Daugherty, A.M.; Haacke, E.M.; Raz, N. The Role of Hippocampal Iron Concentration and Hippocampal Volume in Age-Related Differences in Memory. *Cerebral Cortex* **2013**, *23*, 1533–1541, doi:10.1093/cercor/bhs139.
